# Artificial Development by Reinforcement Learning Can Benefit From Multiple Motivations

**DOI:** 10.3389/frobt.2019.00006

**Published:** 2019-02-14

**Authors:** Günther Palm, Friedhelm Schwenker

**Affiliations:** Institute of Neural Information Processing, Ulm University, Ulm, Germany

**Keywords:** reinforcement learning, multi-objective, actor-critic design, artificial curiosity, artificial cognition, intrinsic motivation

## Abstract

Research on artificial development, reinforcement learning, and intrinsic motivations like curiosity could profit from the recently developed framework of multi-objective reinforcement learning. The combination of these ideas may lead to more realistic artificial models for life-long learning and goal directed behavior in animals and humans.

## Introduction

Reinforcement learning (RL) is a well-established learning paradigm, first consolidated in the book of Sutton and Barto (1998) after the early years of artificial neural networks and machine learning, with strong roots in the mathematics of dynamical programming (Bellman, 1957) and in the early behavioral psychology of Pavlovian conditioning and learning (Rescorla and Wagner, 1972).

In recent years, plausible neural mechanisms for all essential components of RL have been found in the brain, in particular in the basal ganglia, but also in frontal cortical areas, perhaps involved in different versions of RL (Wiering and van Otterlo, 2012), which have been developed not only from a technical, but also from a neuroscientific motivation; overviews are given in Farries and Fairhall (2007), Botvinick et al. (2009), Chater (2009), Maia (2009), Joiner et al. (2017), and Wikenheiser and Schoenbaum (2016).

Also in recent developments of robotics, artificial agents, or artificial life, in particular when the focus is on learning interesting “cognitive” abilities or behaviors or on child-like “artificial development” (Oudeyer et al., 2007), the framework of RL is often used. If it is understood to include its continuous version, actor critic design (Bertsekas and Tsitsiklis, 1996; Prokhorov and Wunsch, 1997) reinforcement learning is a very general approach encompassing applications from Go-playing (Silver et al., 2016) to motor control (Miller et al., 1995; Kretchmara et al., 2001; Todorov, 2004; Schaal and Schweighofer, 2005; Lendaris, 2009; Riedmiller et al., 2009; Wong and Lee, 2010; Little and Sommer, 2011).

Here we are considering RL in the context of robotics or rather of artificial agents that learn to act appropriately in a simulated or real environment. Most often this involves continuous state and action spaces which cannot simply be discretized (Lillicrap et al., 2015). So usually the RL paradigm is combined with a neural network approach to represent the reward predicting function (Sutton and Barto, 1998; Oubbati et al., 2012, 2014; Faußer and Schwenker, 2015).

In this context there are a number of issues that this framework cannot easily accommodate:

1. the learning of several partially incompatible behaviors,
2. the balance between exploration and exploitation,
3. the development and integration of “meta-heurictics” like “curiosity” or “cautiousness,”
4. the problem of finding a “state space” and its partial observability,
5. the simulation of apparently changing strategies in animal behavior.

In reaction to the first issue one might argue that RL is just for one particular behavior, not for the combination of several behaviors; for this one would need to combine several instances of RL. Of course, one could also argue that each animal has just one behavior which maximizes its chance of survival and apparent particular behaviors or motives driving it must be subordinate to this ultimate goal, similarly in economic decision making the ultimate goal is financial utility (money) and it would be irrational to follow other rewards from time to time (as in the fairy tale of *Hans im Gück*). All this has been debated at length (e.g., Simon, 1955, 1991; Tisdell, 1996; Gigerenzer and Selten, 2002; Kahneman, 2003; Dayan and Niv, 2008; Dayan and Seymour, 2009; Glimcher et al., 2009; Chiew and Braver, 2011) leading to considerable doubts in a simple utilitarian view in economy and practically to various approaches extending basic RL, often in a hierarchical fashion (Barto et al., 2004; Botvinick et al., 2009). Even a human or robot Go-player has not only to consider Go strategies, but also (on a lower level) to control his arm movements when taking and placing a piece.

The balance between exploration and exploitation has been widely discussed in classical RL and even before that (e.g., Feldbaum, 1965). It has lead to various, often stochastic, amendments to the original basic method without a convincing general solution that works well in most applications. This problem has also inspired more general approaches in more complex scenarios which add special “meta-objectives” like “curiosity” or “cautiousness” to the RL scheme (perhaps first by Schmidhuber, 1991), which again points toward a multi-objective approach. Recently these ideas are discussed in particular in the context of autonomous “cognitive” agents and their “artificial development” (Weng et al., 2001; Lungarella et al., 2003; Barto et al., 2004; Oudeyer et al., 2007).

In biology and human psychology or sociology it is clear that the state space (i.e., the total relevant state of the world) is far from being observable by the senses of the individual animal or human. It might even be doubted whether there is such a state at all. At least it is often asking too much to assume that the individual possesses a representation of the set or space of all possible states. Such scenarios are even outside the usual relatively broad POMDP (partially observable Markov decision process, see Kaelbling et al., 1996) formalism, so biologically motivated realizations of RL often rest on relatively simple versions of RL that don't require knowledge of a “state” in the sense of physics, but just rely on sensory and reward input.

Also the last issue is clearly at variance with the basic model of classical RL. However, when we consider the creation of artificial autonomous agents or artificial animals an obvious potential answer to all of these issues comes to mind: Such an agent or animal usually has several different, sometimes conflicting goals or motivations (e.g., food, drink, and sex) which cannot simply be combined linearly to form one general objective (Liu et al., 2015).

It therefore seems natural to use different instances of RL on different simplified state spaces, which contain incomplete information on different aspects of the physical state of the world, with different objectives or reward functions in different contexts or situations and somehow select the most important ones to determine the agent's behavior in each concrete situation. This means that one has to consider multiple objectives and their interaction in decision making. This problem is studied by a growing research community under the heading of “multiple objective reinforcement learning” (MORL).

The framework of MORL can be used to address and alleviate the 5 problems mentioned above. In fact, it is directly motivated from problems 1 and 5. The dilemma between exploration and exploitation (problem 2) is greatly alleviated by the simple observation that behavior guided by exploitation of one objective usually can be considered as exploration for all other objectives. The development of meta-heuristics or “intrinsic motivations” (issue 3) can be very useful also in technical applications; for the MORL framework advocated here the point is simply to put intrinsic motivations like curiosity or cautiousness side-by-side with the basic “extrinsic” motivation(s). Concerning the state-space (problem 4), in many practical applications a real “state-space” is unknown or at best partially observable. In this case the best one can do is to obtain a sufficiently rich approximate representation for it based on sensory data and reinforcement signals, and more such signals are certainly better than less for this purpose.

## Representing the State Space

In order to obtain an approximate state representation by learning from experience, one can use a neural network, typically a multilayer perceptron (MLP) or “deep network” or methods of reservoir computing (Maass et al., 2002; Jaeger and Haas, 2004) for continuous temporal dynamics, or a combination of both. In complex control problems (Koprinkova-Hristova and Palm, 2010) such a representation is often called a “forward model.” So the agent (biological or artificial) tries to learn a “state representation network,” i.e., a (typically recurrent) network that predicts the next state from a representation of the current state, which integrates sensory input information over time and can be used as input to the evaluation or critic network in the usual situation where the current sensory input is insufficient to determine the “state” of the environment; see for example (Sutton and Barto, 1981; Schmidhuber, 1991; Dayan and Sejnowski, 1996; Herrmann et al., 2000; Gläscher et al., 2010). Such a network can be used as the basis for a second network representing the quality or value function in reinforcement learning or actor-critic design.

The use of neural networks or parameterized approximators as estimators of the state-value or state-action-value function is a way to deal with large or continuous action and state spaces. The approximating function may be a linear or nonlinear function of their parameters, but linear approximators show limitations in their expressive power, while convergence of learning is quaranteed. Nonlinear approximators, typically neural networks, are universal approximators (Cybenko, 1989), but often show instable behavior during learning. During the last years increasingly complex networks are used in RL for large and continuous state spaces; in addition to classical multilayer perceptrons or radial basis function networks, also trainable recurrent neural networks (Hagenbuchner et al., 2017) or echo-state-networks (Scherer et al., 2008; Oubbati et al., 2012, 2013, 2014; Koprinkova-Hristova et al., 2013) are used, and particular methods have been developed to improve the stability of learning (Hafner and Riedmiller, 2011; Silver et al., 2014; Faußer and Schwenker, 2015; Lillicrap et al., 2015; Parisi et al., 2017). Recently, deep neural networks such as autoencoders and convolutional neural networks have been applied for representation learning and used in combination with RL methods to learn complex decision task from raw data (Lillicrap et al., 2015; Mnih et al., 2015; Mossalam et al., 2016; Srinivasan et al., 2018).

In any case it is practically important for MORL to use one and the same network as a basis to create a sufficiently rich representation in order to train all different objectives (critics and actors) as outputs of the last layer (Mossalam et al., 2016).

Based on the sensory input alone, but also on such an approximate state representation, it often will not be possible to predict the expected reward or the next state with certainty. In a neural network for classification, for example, this uncertainty will be expressed by submaximal activation of several output neurons and these activations may be interpreted as *a posteriori* probabilities of the various outcomes (states or values); the uncertainty in estimating the expected reward is often measured by its variance. Beyond variance, there are various formalisms for calculating measures of certainty or uncertainty from these probabilities, often in terms of information theory (Palm, 2012), and several approaches to incorporate measures of uncertainty, or of “novelty” or “surprise” into the choice of appropriate actions in reinforcement learning (e.g., MacKay, 1992; Sporns and Pegors, 2003; Little and Sommer, 2011; Tishby and Polani, 2011; Sledge and Pŕıncipe, 2017); much of this is reviewed and discussed by Schmidhuber (1997) or Schmidhuber (2003) also in relation to the exploration-exploitation dilemma (Dayan and Sejnowski, 1996; Auer, 2002; Tokic and Palm, 2012; Tokic et al., 2013). Again these practically important considerations point toward MORL, for example in the direction of additional “meta-objectives” like curiosity or cautiousness (Wiering and Schmidhuber, 1998; Uchibe and Doya, 2008; Oubbati et al., 2013). It is often useful to consider at least two versions of the primary objective, namely its expected value and an estimate of the value that can at least be obtained with a reasonably high probability (e.g., the 5-percentile).

The MORL idea transforms the original problem of learning one behavior that is useful in all circumstances into a problem of designing an appropriate architecture for learning and decision making that combines several (probably hierarchically organized) instances or stages of classical RL and possibly other methods of learning or decision making (Oubbati and Palm, 2010).

## Multi-Objective Reinforcement Learning

A framework for studying these problems in the restricted realm of reinforcement learning, which has recently gained increasing popularity, is called MORL (see Roijers et al., 2013; Liu et al., 2015). We would like to propose to use this framework as a starting point to tackle the broader architectural problem in some concrete scenarios, which occur quite naturally in many technical optimization and control problems and have been elaborated in the MORL community, some examples (Deep Sea Treasure, Bonas World, Cart Pole, Water Reservoir, Resource Gathering, Predator Prey) are described in Drugan et al. (2017) and the literature cited therein; see also Vamplew et al. (2011).

The difference of MORL to classical RL is quite simple: If we think in terms of actor-critic design, where essentially an evaluation of the agent's actions is learned in a POMDP and where this evaluation function may be learned by a neural network, now we just have a vector of evaluations instead of a single value (in the output layer of the network). Similarly there is now an actor for each component of the evaluation vector suggesting an appropriate action for that particular value, objective, or motive. This model clearly leads to the problem how to combine the different objectives and suggested actions in order to decide on the next action. This problem has been discussed thoroughly in the MORL community; for an overview see Liu et al. (2015) and Drugan et al. (2017) and we will contribute a few ideas on this issue in terms of the computational architecture. The most common idea is to combine the different reward values into a weighted sum and take the best action for this combination. More complex methods consider the so-called *pareto-front*, well-known from classical multi-objective optimization. In fact, much of the discussion on optimal decision making for multiple objectives and methods for finding the pareto-optimal solutions (Das and Dennis, 1998; Miettinen, 1999; Mueller-Gritschneder et al., 2009; Motta et al., 2012) can be useful for MORL (see Van Moffaert and Nowé, 2014; Pirotta et al., 2015; Vamplew et al., 2017).

Once the most appropriate action has been determined and carried out, each of the actors and critics is able to learn something from its outcome leading to a modification of the corresponding neural networks, usually through backpropagation of the expected reward update or temporal difference.

From introspection, but also from behavioral animal experiments one gets the impression that each of these motives enters the final evaluation and decision with its own weight or “urgency” that may vary with time, depending on the agent's needs, which implies that there is no fixed “trading relation” between the different motives and their corresponding reward values, so they cannot be reduced to just one value. Modeling artificial agents in this wider framework entails some new problems and tasks, which may also lead to new interesting research projects and interactions with behavioral biologists and psychologists.

Here we describe the basic theoretical framework for this approach:

1. Given *n* motives, *n* current predicted values (*v*_1_, …, *v*_*n*_), and *n* “urgency weights” (*w*_1_, …, *w*_*n*_) for them, how do we combine them to one value that should be maximized by the next action? There are different more or less obvious ideas for this (see e.g., Boutilier, 2002; Castelletti et al., 2002; Natarajan and Tadepalli, 2005; Wiering and De Jong, 2007) also motivated by modeling animal behavior, or reflecting the introspective difference between positive and negative rewards, or between goal seeking and pain avoidance, the most obvious and simple being the weighted sum $v=\underset{i}{\sum }w_{i}v_{i}$. At the opposite extreme we would follow the one objective that has maximal *w*_*i*_*v*_*i*_, or we could consider a minimal value for some objectives as a constraint in maximizing the weighted sum of the others. Here the “higher” motives like curiosity are put side-by-side with “lower” ones like “hunger,” which may be psychologically somewhat unsettling, but might actually work. We first encountered this idea in the work of Dörner (2001), see also Bach (2009) and Bach (2012).
2. For each of the motives, in addition to defining the corresponding rewards *r*_*i*_ we have to model their “urgency function” *w*_*i*_(*t*). This may involve a dynamical system model of the agent's body and as such may be considered as part of the world model. In particular, it will use the corresponding rewards *r*_*i*_(*t*) as inputs. In extreme cases *w*_*i*_ may even be constant or it may simply integrate the incoming rewards as
  $${ẇ}_{i}\left(t\right)=a-br_{i}\left(t\right)\;\text{or}\;\tau {ẇ}_{i}\left(t\right)=-w_{i}\left(t\right)-br_{i}\left(t\right)+a$$
  but much more is easily conceivable, for instance involving thresholds at which the urgency changes drastically. The development of such dynamical models of urgency may be an interesting line of research also in modeling animal behavior. Actually, the simple integration model was probably first introduced informally by Lorenz (1978).
3. It is now possible to introduce some more “cognitive” motives like “curiosity” (see also Pisula, 2009), for which we have to define *r*_*i*_(*t*) and *w*_*i*_(*t*). For example for curiosity it is natural to define surprising events as rewarding, where surprise may be defined as −log*p* relative to a probabilistic world model that the agent may have learnt (Palm, 2012). More concretely, if in world state *x* the agent receives the observation *o*(*x*), or the state description *d*(*x*) (Palm, 2012), which has the probability *p*(*x*) = *p*(*d*(*x*)) in his current model, then his surprise is −log*p*(*x*). Then again *w*_*i*_(*t*) can be defined for example by an integration model.
4. Finally we have to decide for the optimal action. Given our estimates for the temporal rewards and urgencies of the different motives and also our momentary combined reward, we can use methods of multi-objective or of plain optimization to find the optimal action. As a starting point we can use the actor outputs for the individual motives and perhaps try their combinations. Practical methods for finding a reasonable solution to the optimization problem in short time are also discussed in the literature on RL and MORL (Handa, 2009; Kooijman et al., 2015; Brys et al., 2017; Parisi et al., 2017; Vamplew et al., 2017).

This leads to an extended RL-architecture, which may be biologically more realistic. Such a more complex architecture also offers interesting additional possibilities for improving behaviors by learning: The existence of more objectives compared to just one, generates a richer representation of (the value of) the current situation, which can be used also to improve the sensory-based world model. It also gives a new perspective on the exploration-exploitation dilemma, since following exploitation of one objective may serve as exploration of the others. We have presented a basic layout of such a multi-objective agent architecture and started some preliminary experiments on it (Oubbati et al., 2013, 2014), but we believe that much more can and should be done in this direction.

## Author Contributions

GP: involved in preparing the concept of the paper, and writing of the paper; FS: writing of paper including literature work and proofreading.

### Conflict of Interest Statement

The authors declare that the research was conducted in the absence of any commercial or financial relationships that could be construed as a potential conflict of interest.
